# Explanatory models in real-world clinical interactions at a pediatric cancer center in Guatemala

**DOI:** 10.1371/journal.pgph.0003813

**Published:** 2024-10-11

**Authors:** Laura C. Harrison, Silvia Rivas, Lucia Fuentes, Ana Cáceres-Serrano, Gia Ferrara, Federico Antillon-Klussmann, Carlos Rodriguez-Galindo, Jennifer W. Mack, Dylan E. Graetz

**Affiliations:** 1 Department of Pediatrics, University of Tennessee Health Science Center, Memphis, Tennessee, United States of America; 2 Department of Palliative Care, Unidad Nacional de Oncología Pediátrica, Guatemala City, Guatemala; 3 Department of Psychology, Unidad Nacional de Oncología Pediátrica, Guatemala City, Guatemala; 4 Department of Global Pediatric Medicine, St. Jude Children’s Research Hospital, Memphis, Tennessee, United States of America; 5 Department of Oncology, Unidad Nacional de Oncología Pediátrica, Guatemala City, Guatemala; 6 Department of Oncology, Francisco Marroquin University School of Medicine, Guatemala City, Guatemala; 7 Departments of Pediatric Oncology and Population Sciences, Dana Farber Cancer Institute/Boston Children’s Hospital, Boston, Massachusetts, United States of America; University of Embu, KENYA

## Abstract

Explanatory models (EMs) are used in medical anthropology to characterize individual understandings of illness. This study investigated how interdisciplinary clinical interactions elicited caregiver EMs at a pediatric cancer center in Guatemala. This qualitative study included caregivers of 20 children with newly diagnosed cancer at Unidad Nacional de Oncología Pediátrica (UNOP) in Guatemala City, Guatemala. UNOP’s diagnostic process includes social work intake, psychoeducation with a psychologist, and a diagnostic conversation with an oncologist and psychologist. Audio-recordings from the diagnostic process and a semi-structured interview were obtained, transcribed, and translated from Spanish. Transcripts were coded using a priori codes based on the five explanatory model (EM) components (*occurrence*, *causation*, *pathophysiology*, *course of sickness*, and *treatment)*, as well as *disease*, and *illness accounts*. Thematic content analysis explored the EM framework as applied to diagnostic interactions between families and clinicians. All five components of the EM were addressed during the diagnostic process at UNOP. Clinicians, particularly psychologists, initiated conversation about the EM more than caregivers. When prompted, caregivers discussed all aspects of the EM but only rarely mentioned *pathophysiology*. *Disease accounts* were primarily described by clinicians, while caregivers used *illness accounts* to describe cancer *causation*. Clinicians validated existence of both *disease* and *illness accounts*. UNOP’s interdisciplinary team elicited families’ beliefs and facilitated in-depth discussion of all aspects of the EM, leading to a shared understanding of cancer and its treatment. Utilizing the EM framework in clinical practice may support culturally-competent pediatric cancer care.

## Introduction

Beliefs and cultural context have been increasingly recognized as integral to patients’ interaction with the healthcare system [1]. In medical anthropology, a distinction is made between *disease*, which describes biologic or pathologic processes, and *illness*, which is a personal or cultural reaction to disease or discomfort [2]. Whereas clinicians are historically taught to treat disease, patients experience illnesses. Arthur Kleinman characterized an individual’s notion of illness through the concept of explanatory models (EMs), which includes beliefs around *occurrence*, *causation*, *pathophysiology*, *course of sickness*, and *treatment* [2]. An individual’s explanatory model (EM) influences their understanding of symptoms and engagement with treatment [3–6].

In an effort to address health disparities and improve outcomes, there has been increased emphasis on the patient-clinician interaction and patient-centered care [7–10]. EMs are influenced by social and cultural belief systems, and thus clinicians’ EMs often differ from those of their patients [3, 11–18]. Conflicting clinician and patient EMs can result in moral distress, miscommunication, suboptimal adherence, and poorer outcomes [19, 20]. To address these issues, Kleinman advocated for training health professionals in anthropologic concepts with a focus on addressing both *disease* and *illness* [2, 21].

Patient EMs of cancer can influence understanding of risk, participation in screening, and adherence to treatment [1, 22]. Treatment abandonment may be influenced by myths around cancer origin and contributes to disparities in pediatric cancer outcomes, especially in low- and middle-income countries where 80% of children with cancer live [23–25]. Communication strategies within pediatric oncology have been studied in an effort to improve patient-centered care and outcomes [23–27].

In this study, we investigate how providers elicited and engaged with caregiver EMs in real-life clinical encounters at Unidad Nacional de Oncología Pediátrica (UNOP), a pediatric cancer center in Guatemala. Recognizing the need to address treatment abandonment, in 2005 UNOP implemented an interdisciplinary team, consisting of psychologists, social workers, palliative care providers, and child life specialists to provide educational sessions, address knowledge gaps, and support families from diagnosis through therapy completion [28]. We hypothesize that all components of the EM were addressed through sessions conducted by this team and explore the ways in which these interactions shaped caregivers’ understanding of cancer and its treatment.

## Methods

### Setting and participants

This study was conducted in Guatemala City, Guatemala at UNOP, Guatemala’s national pediatric cancer center. Spanish-speaking caregivers of children ≤18 years of age with a new or likely diagnosis of cancer were eligible. Initially, the research team approached all caregivers of newly diagnosed children sequentially and offered study participation. Purposive sampling was also done as needed to include patients of varied socioeconomic classes, ages, and diagnoses. Thematic saturation was achieved at a sample size of twenty families [29]. Thirty-two families were approached prior to saturation; twelve declined participation. The most common reason for declining participation was hesitancy about being audio-recorded; one had a language barrier. The children of caregivers who declined had similar ages, genders, and diagnoses to those who participated. Data was collected April 10 through August 23, 2019. All participants provided written informed consent. The study was approved by the Institutional Review Boards at St. Jude and regulatory committee at UNOP. Details of this study’s secondary analysis are provided below; full methodology for the original study has previously been published [30].

### Data collection

UNOP’s standard intake process begins with collection of demographic information by a social worker. Next, families meet with a psychologist for psychoeducation, in which they are asked additional questions and provided cancer education. Finally, there is a diagnostic conversation with an oncologist and the psychologist who had been present during psychoeducation to discuss their child’s diagnosis and treatment plan. For this study, psychoeducation and diagnostic conversations, referred to collectively as the diagnostic process, were recorded. Following diagnosis, audio-recorded semi-structured interviews with caregivers explored their experiences with the diagnostic process. An interview guide was utilized for semi-structured interviews that explored families’ experience with the diagnostic process, as well as their understanding of cancer (S1 File). Interviews were conducted by local study team members with training in psychology and qualitative methods who were employed by UNOP but not directly involved in the care of the participants they interviewed. All recordings were professionally transcribed and translated into English. Translations were reviewed by bilingual members of the research team to ensure accuracy and data integrity.

### Data analysis

Demographic information was tabulated using descriptive statistics. Thematic content analysis was performed using a priori codes based on Kleinman’s five components of the EM, including *occurrence*, *causation*, *pathophysiology*, *course of sickness*, and *treatment*, as well as Kleinman’s concepts of *disease* and *illness accounts* [2]. Code definitions can be found in Table 1. Each transcript was independently coded by two members of the research team (DG, GF) using MAXQDA software [31]. Disagreements between coders were resolved through consensus and with a third-party adjudicator (JM) as necessary. All three authors involved in coding are US based qualitative experts, two of whom are oncologists and one of whom is fluent in Spanish. Segments identified using codes were reviewed using constant comparative analysis conducted primarily by LH, a US based pediatrician, with reflexive review by all members of the research team [32, 33].

**Table 1:** Definition of codes utilized for analysis.

Codes	Definition
Occurrence	Descriptions of the occurrence of the disease; when it was first noticed.
Causation	Descriptions of cancer causation, including caregivers asking about origin.
Pathophysiology	Descriptions of disordered physiologic processes associated with cancer/disease including how the disease works in the body.
Course of sickness	Descriptions of sickness course including severity and length of course. Includes when the provider is generally describing the course of cancer development and specific references to the patient, as well as descriptions from the caregiver or provider about the progression of the disease itself.
Treatment	Descriptions of treatment (specific to cancer) for the child’s illness including recommendations for treatment and questions about treatment, including potential treatment modalities: chemotherapy, surgery, radiation. Also includes references to treatment length and other general discussion of treatment.
Disease account	Descriptions (particularly of etiology) that focus on physical aspects of patient as source of sickness.
Illness account	Description (particularly of etiology) focusing on person, social environment, or universe at large as source of sickness and place of occurrence.

## Results

Twenty families (*n* = 40 caregivers) participated in this study. Demographic information for the caregiver participants and clinicians is presented in Tables 2 and 3. All five components of the EM were identified throughout the recorded conversations and interviews. Overall, discussions about the EM were largely prompted by clinicians. Spontaneous mention of EM components by caregivers was less common but they had much to say when asked. Each component of the EM is discussed in more detail below (Table 4).

**Table 2:** Participant characteristics *n* (%).

Patient diagnosis	
Acute Lymphoblastic Leukemia	11 (55)
Acute Myeloid Leukemia	2 (10)
Burkitt Lymphoma	1 (5)
Hodgkin’s Lymphoma	1 (5)
Wilms tumor	2 (10)
Osteosarcoma	1 (5)
Histiocytosis	1 (5)
High-grade glioma	1 (5)
Religion	
Evangelical	13 (65)
Catholic	4 (20)
Other	3 (15)
Mother’s education	
No school	6 (30)
Elementary school	8 (40)
More than elementary school	1 (5)
Unknown	5 (25)
Father’s education	
No school	2 (10)
Elementary school	15 (75)
More than elementary school	3 (15)
Mother’s occupation	
Housewife	18 (90)
Day worker	1 (5)
Professional	1 (5)
Father’s occupation	
Unemployed	1 (5)
Day worker	1 (5)
Farmer	9 (45)
Professional	8 (40)
Unknown	1 (5)
Caregiver present for psychoeducation	
Mother	9 (45)
Father	5 (25)
Both	6 (30)
Caregiver present for diagnostic conversation	
Mother	7 (35)
Father	5 (25)
Both	8 (40)
Caregiver interviewed	
Mother	13 (65)
Father	7 (35)

**Table 3:** Clinician demographics *n* (%).

Profession	
Pediatric hematologist/oncologist	7 (70)
Psychologist	3 (30)
Age (years)	
<30	2 (20)
30–49	5 (50)
>49	3 (30)
Gender	
Female	6 (60)
Male	4 (40)
Years of clinical experience	
1–3	4 (40)
4–9	3 (30)
10+	3 (30)

**Table 4:** Explanatory models of pediatric cancer among caregivers and clinicians at UNOP.

	Caregiver	Clinician	Key insights
**Occurrence**	She started with throat pain and stopped eating because the pain was too much. (psychoeducation) I noticed something was not right, she didn’t want to play much (psychoeducation) I went to the doctor; he gave us medicine, antibiotics, but she wasn’t recovering. (interview)	Start telling me when [your son] started to feel bad, what symptoms did he have, and how did you get here. (psychologist, psychoeducation) Finally, can you please tell me a little bit about [your daughter’s] medical history. When did this start, what were her symptoms and what did you do, all the road you walked until you got here. (psychologist, psychoeducation)	Clinicians, usually psychologists, asked about initial symptoms. Caregivers noted either changes in behavior or symptoms as signs that their children were potentially ill.
**Causation**	At home we’re always washing the dishes and avoiding the flies that are everywhere, and we see those people in the street eating garbage and they are fine. (psychoeducation) It’s so difficult to understand why theses illness exist. Because I say, I’ve given her good food, I’ve taken good care of her, then why is this happening? (psychoeducation) This was recently, after the fall, was this a result of the fall? (psychoeducation) Last year my mother’s dog bit our son, so my husband said maybe he’s got fear. … But I told him I never knew a case like that, I’ve never heard that fear could cause something like this, I’ve heard of diabetes. I told him I didn’t think that was the cause. (diagnostic conversation) I wondered where it came from, they said it was a cell that started to grow, and it was an enemy for the white cells and started to kill them all. (interview) Well, they told me this happens as an accident. It’s not because I couldn’t take care of her. That this illness comes without warning, it’s not a result of lack of treatment. (interview)	So many parents ask me, why does my son have cancer? (psychologist, psychoeducation) What I want you to keep in your heart…is that is not your fault, is not her dad’s fault and is not [your daughter’s] fault either. There is nothing that you could do or stop doing so this doesn’t happen; it’s just something that happens. (psychologist, psychoeducation) Here at the hospital, we have children that live in better conditions than others, children that have a high level of hygiene and children that do not, and they both have cancer. It’s not because of sadness or bad temper, is not because of *susto*, that’s a completely different illness…If you want to see it this way, cancer is like an accident, it’s something that happens and we don’t know why, but we know how to treat it, and that’s the important thing. (psychologist, psychoeducation) Look, very often parents tell, is it due to bad caring? Or as you felt, it’s because I didn’t find out on time, or maybe they were hit, maybe something he ate, but in fact we haven’t discovered what’s happening with these illnesses, or where they come from. (psychologist, diagnostic conversation) This could happen to any child. Undernourished children, children with a good diet, black or white children, any person. This is not the parent’s fault nor the child’s fault (oncologist, diagnostic conversation)	Caregivers related cancer to blame or the cultural belief of *susto*. In interviews, caregivers reflected new understanding around cancer *causation*. Clinicians dispelled common cultural myths about cancer etiology.
**Pathophysiology**	I know it’s a deformation, a mutation of the cells, the bad ones. They don’t die, instead they infect other good cells. (psychoeducation) what I understand is that the cancer is… I don’t know… but I suppose the type she has is very… very violent, how to say, aggressive, it extends to other cells. (interview) I wondered where it came from, they said it was a cell that started to grow, and it was an enemy for the white cells and started to kill them all. (interview)	Cancer is also a cell, but this is a bad cell, it’s a cell that harms the body instead of helping it, because it has not functionality, no job…This cell has no purpose, so instead of helping, it harms the body. (psychologist, psychoeducation) Defenses, like the name implies, defenses are the police of our body, they defend me from the flu, fever, infections…Yes, there are a lot of police men but they are not doing their job, they are disorganized (psychologist, psychoeducation) This tumor grows like if it has a root, it’s very deep in the brain, even if radiotherapy kills it, most of the time the root is still there, bad cells in the good parts of the brain, that’s why it grows again, and since those are very small things that can’t be seen easily, even with tomography or resonance, we can’t see if there are cells inside the brain. (oncologist, diagnostic conversation)	Caregivers rarely brought up *pathophysiology*. Psychologists utilized metaphors to explain the *pathophysiology* of cancer.
**Course of sickness**	They gave him vitamins, and then he started to get pale. (psychoeducation) I think he’s better than before when I brought him here, because now he’s eating, he wasn’t eating before. His nose is not bleeding anymore, nor is his gums. (diagnostic conversation) This is all a result of the illness, now she’s feeling better, she’s eating and drinking normally. I see her better. She’s calmed. She’s not living the nightmare anymore. She’s eating well, and I think the pills are helping her, I don’t know what they are but they give those pills with food, so I guess that’s helping. (interview)	We must know that this is not forever, the treatment has a beginning and an end. It’s a sacrifice, and if she has cancer, it’s worth fighting for. (psychologist, psychoeducation) Why is the treatment this long? Because leukemia may come back, so we kill it little by little but this is a smart illness, it can hide for a while and when it doesn’t feel the chemo then it comes back (oncologist, diagnostic conversation) And when we say cure, we mean they are going to grow up, be adults, have children, they’ll have a normal life. (oncologist, diagnostic conversation) In week 7…we’ll see how is the hemoglobin, the skin, the ganglions…the ideal should be if they have disappeared. (oncologist, diagnostic conversation)	Caregivers tracked illness progression by the child’s return to typical activities. Clinicians mirrored the language used by parents to describe clinical course.
**Treatment**	First of all, we asked if there was a cure for that or we would have to take him home and watch him die. (psychoeducation) Many people got me scared because they said that for this there was no cure and no medicines. I used to pray to God for help, and other people said to me that there was medicine for this (psychoeducation) One last question, about the chemotherapies, you told us some are injected, some are drinkable…I imagined something else…I imagine electric shocks. (diagnostic conversation) They explained to me and my husband the problem she had in her blood and told us that the treatment was going to be long but that here she will receive everything. Thank God she is receiving the right treatment, she has a different mood now, because she was really down, she had no physical activity but know I see a change in her. (interview) He told me it was a disease in the blood, that were little balls walking through the body and that’s why my baby was going to receive treatment so the little balls couldn’t reach his brain. (interview)	We don’t know why it’s happening, what we know is the way to treat it. (psychologist, psychoeducation) When I receive chemotherapy, I could have infections or diarrhea because let’s say police guards in the body will be sleeping, so if I eat a fried chicken at the street, it won’t harm me because my guards are defending me, but if she eats that, she can have a stomach infection or diarrhea, because her guards will be sleeping. (psychologist, diagnostic conversation) Part of the treatment is chemotherapy; chemotherapy is the medicine for cancer, just like there is medicine for the flu, medicine for ear infection; there is medicine for cancer. (oncologist, diagnostic conversation) We are not able to cure all patients, nowhere in the world oncologists are able to do that, but there is a possibility and that’s what we want to offer you today, treatment. But this means that we must get through the entire process of treatment and that’s what we wanted to talk with you. I want you to stick to that possibility, with the help of God she will be cured. (oncologist, diagnostic conversation)	*Treatment* was the most discussed EM component. Caregivers had varied understanding of cancer *treatment*. Psychologists used metaphors to explain chemotherapy.

### Occurrence

Disease *occurrence* was most frequently discussed during psychoeducation, and when it was described by caregivers, it was typically prompted by psychologists. After asking demographic questions, psychologists asked caregivers to describe how they noticed their child was ill. Some caregivers noticed symptoms; others discovered masses or lesions during routine childcare, “I always change my baby…I touched his hair and I felt a little ball on the back of his ears” (caregiver, psychoeducation). For others, a change in their child’s behaviors caused them to seek care, “Suddenly…she didn’t want to play anymore…she was like bored and I got worried” (caregiver, interview). *Occurrence* came up less frequently during diagnostic conversations, as oncologists began with questions about initial interactions with the healthcare system, “What did the doctors tell you at the other hospital before they sent you here?” (oncologist, diagnostic conversation).

### Causation

*Causation* was also primarily discussed in psychoeducation encounters. Some caregivers brought up causation when asked what they knew about cancer, “It’s so difficult to understand why theses illness exist…I’ve given her good food, I’ve taken good care of her, then why is this happening?” (caregiver, psychoeducation). Clinicians frequently initiated conversation around *causation*, “When caregivers come here for the first time, they ask me two questions…I want to know what is cancer and why it happens” (psychologist, psychoeducation). They used *causation* as a prompt before providing an explanation of cancer and discussion about what is known and unknown about the causes.

Caregivers wondered why their child got cancer and offered up a myriad of possible explanations unprompted. Many caregivers posited explanations that centered around blame, “​​I always asked myself…have we been careless” (caregiver, interview). Several caregivers referenced *susto*, a cultural belief that medical conditions result from a terrifying event, “Last year my mother’s dog bit our son, so my husband said maybe he’s got *susto*” (caregiver, diagnostic conversation) [34]. Others who were unable to find an explanation for their child’s cancer relied on faith to help them manage uncertainty, “God knows why these things happen” (caregiver, interview).

Whether or not caregivers offered up their own explanations of *causation*, clinicians dispelled common cultural myths surrounding cancer etiology and emphasized the random and accidental occurrence of cancer, “It has nothing to do with hygiene or bad nutrition, it’s not a result of something you gave him or a flu that was not treated or anemia…this is not your fault…You may see this as an accident, this is something that happens and we don’t know why. It’s not evil eye or *susto*” (psychologist, psychoeducation). Caregivers demonstrated internalization of this concept during interviews, “She said it’s not hereditary, that it’s nobody’s fault, it just appeared” (interview).

### Pathophysiology

The *pathophysiology* of cancer was predominantly discussed by clinicians and rarely mentioned spontaneously by caregivers. Psychologists spent the most time explaining *pathophysiology*. During psychoeducation, psychologists asked caregivers what they knew of cancer and educated them about cancer *pathophysiology*. To aid in their explanations, psychologists used metaphors, “A bad cell was born within the policemen, the white blood cells. And this bad cell grew, but it reproduced too fast…it invaded the blood factory and it’s not working properly anymore” (psychologist, psychoeducation). Many employed culturally relatable metaphors, such as imagery associated with agriculture, “cancer doesn’t stay in one place, it’s like a bug that sends seeds to other parts of our body” (psychologist, diagnostic conversation). Alternatively, oncologists tended to use allopathic medical terminology to describe cancer *pathophysiology*, “There are blood cells called lymphocytes that are part of our immune system, and at some point, in the natural growing process of the cell they transform from normal to malign” (oncologist, diagnostic conversation).

Caregivers said little about *pathophysiology* during the diagnostic process but sometimes reflected on this concept when prompted in interviews. In these cases, caregivers utilized the language that clinicians had used during their explanations; “I am a farmer, so, they told me it was like when I sow and a poisonous animal kills my plant, they told me to think it like that, that’s the baby’s life, a cell wanted to kill him” (caregiver, interview).

### Course of sickness

*Course of sickness* was discussed throughout the encounters as families were asked about care prior to arriving at UNOP. As UNOP is a tertiary care center, families often arrived there only after initial attempts at treatment did not work. Some children were seen by traditional healers, “She was very down, so all people started saying it was evil eye and *susto*, so we took her to the healer. Then, we saw she was not getting better so we decided to take her to the hospital” (caregiver, psychoeducation), while others had been evaluated by allopathic providers. When describing initial work-up, caregivers used terminology given to them by the providers their children saw, “They told us he had a problem with the blood vessel and his liver” (caregiver, psychoeducation).

Caregivers often tracked improvement of disease as it related to their child returning to normal activity, “If he wasn’t improving, he wouldn’t eat, he wouldn’t play, he wouldn’t walk; but since he is eating, walking and playing, that’s a sign he is fine” (caregiver, interview). Many clinicians adopted similar language when discussing response to treatment, “So, they get used to the medicine, they start feeling better, they change their attitude, they start going out of bed” (oncologist, diagnostic conversation), or describing the path to cure, “We have children that had this illness and now they are all grown up, they have their own children” (psychologist, psychoeducation). Clinicians also emphasized to caregivers that even without symptoms, cancer may still be present, “Even though she has no visible symptoms, her blood is not working well” (psychologist, psychoeducation), stressing the importance of continued evaluation and adherence to treatment. Caregivers responded with understanding of this message; “We must always come… We’ll try to be very responsible for him” (caregiver, diagnostic conversation).

### Treatment

*Treatment* was the most frequently discussed EM component. It was introduced during psychoeducation and further reviewed in detail during diagnostic conversations. During psychoeducation, some caregivers brought up *treatment* in response to being asked about cancer; “Well, I’ve heard it’s curable. That there is also a special treatment. That it takes time, but there’s a cure” (caregiver, psychoeducation). Caregivers were also asked if they had heard of chemotherapy. Some caregivers had a limited understanding of chemotherapy, “That is something, little bottles, that are injected in the vein or something, and that because the chemical is hot…the hairs start to fall off, or the nails look different” (caregiver, psychoeducation), while others were unfamiliar with cancer treatment. To describe chemotherapy and its side effects, psychologists again used metaphors, “What does chemotherapy do? It kills cancer cells, but…chemotherapy is like a baby, when babies are born, do they know what is good and bad or do they learn it?…They don’t know. Chemotherapy sometimes confuses good cells and bad cells” (psychologist, psychoeducation). Oncologists often framed conversations about treatment optimistically, “Today we can cure cancer,” before delving into the details of treatment plans, “treatment will require him to take some syrups, pills, or saline solution…called chemotherapy, and they are special medicines to clean the blood” (oncologist, diagnostic conversation). In interviews, caregivers were able to reiterate these explanations of chemotherapy; “She said cancer is a bunch of bad cells, but chemo will destroy the cells, even the good ones and that’s why he loses hair, is nauseous and feels down; but that’s the only treatment” (caregiver, interview).

### Disease and illness accounts

As clinicians and caregivers described components of the EM, both *disease* and *illness accounts* emerged (Table 5). *Disease accounts* refer to biologic or pathologic explanations of a medical condition, while *illness accounts* describe the cultural or personal experience of symptoms.

**Table 5:** Caregiver and clinician disease and illness accounts of cancer.

	Caregiver	Clinician	Key Insights
**Disease Account**	I know it’s a deformation, a mutation of the cells, the bad ones. They don’t die, instead they infect other good cells. (psychoeducation) She told us that based on the results from the peripheric rub, that’s a blood test on a lamella, where they saw the cells, they detected some bad cells. That these cells are progressing very fast and that they are contaminating the rest of the cells. (psychoeducation) Well, the doctor told us that in the peripheric rub they performed in him, they found bad cells, they maybe be leukemia cells, cancer cells. (diagnostic conversation) At the beginning they told me it was a mass; they call it tumor. (interview)	Cancer is also a cell, but it’s a bad one, it’s like that bug that penetrates my crops. It contaminates my crops. Cancer, instead of helping the body, it harms it. And cancer, just like the good cell, has a process of life. A bad cell is born, it grows, but what’s the problem? It reproduces too fast. (psychologist, psychoeducation) Where is her leukemia? Her leukemia right now is all over her body, it’s now circulating through her whole body and we must kill, little by little, this leukemia. (oncologist, diagnostic conversation) What happens with leukemia is that when the cell is formed, when it is a baby cell let’s say, and in its growing process, there’s a moment when it suffers a damage and it starts reproducing more and more. That’s leukemia. (oncologist, diagnostic conversation)	Clinicians were more likely to use *disease accounts* than caregivers. When caregivers used *disease accounts*, it was in reference to what other providers had explained to them about their child’s symptoms.
**Illness Account **	Well, honestly, they told me that he had got the eye, and frankly I don’t believe in that stuff because I had my older sons and none of them got that, so I said that doesn’t exist. (psychoeducation) She knows it’s malign, but she believes in God and she thinks God will cure her, will cure her head. She’s also evangelic and she prays a lot and she believes in God will help her overcome this. (psychoeducation) She loved eggs and lemon…but she used to eat too much lemon. I blame the lemon. (psychoeducation) And they started telling her stuff like she was going to get *susto* and things like that…and suggested a witchdoctor to perform some sort of a ritual with grass and stuff. And this is why it took us longer to bring her (diagnostic conversation)	We know it can hit any child, at any age, it’s not due to a lack of vitamins, or food, it’s not because of something that happened during pregnancy, or due to *susto* or evil eye. (psychologist, psychoeducation) And like you said…there are other types of illnesses, [such as] evil eye and these illness are treated in different ways. We have had patients here that say, we cure the evil eye and nothing, that’s because it was not that illness. (psychologist, psychoeducation) We have one girl who has cancer and was cure of fear at the same time she was taking her cancer medicine. They are different illnesses. (psychologist, psychoeducation) This is something that God allowed to happen, and he can also remove it. (oncologist, diagnostic conversation)	Caregivers used *illness accounts* to explain cancer causation. Psychologists acknowledged that *illness* and *disease accounts* can co-exist.

*Disease accounts* were utilized more frequently by clinicians than caregivers, specifically when explaining *causation*, *course of illness*, or *pathophysiology*, “We suspect that a bad cell, we don’t know why, was born in the body police, the defenses. But it grew and started to reproduce too fast, to the point that invaded the blood factory and that doesn’t allow it to work properly” (psychologist, psychoeducation). Caregivers did not commonly use *disease accounts*. When they did, it occurred primarily in the interviews when they were asked how they understood cancer. In response, caregivers often reiterated what another provider had relayed to them, “He told me it was a disease in the blood, that there were little balls walking through the body and that’s why my baby was going to receive treatment” (caregiver, interview).

*Illness accounts* were utilized by both caregivers and clinicians. Caregivers used *illness accounts* to explain beliefs they held regarding why their child was sick, “So, one person told us he was suffering from *susto* and other stuff. And she told us, let’s cure him before you get him to the hospital. So, they bathed him, they did all the ritual required…then she told us…you must take him to the hospital, he’s really sick” (caregiver, psychoeducation). Almost all *illness accounts* related to the concept of “susto.” Psychologists were more likely to utilize *illness accounts* than oncologists; acknowledging and validating the coexistence of *illness* and *disease accounts* to explain a child’s symptoms, “​​It’s not because of sadness or bad temper, is not because of *susto*…*Susto* and leukemia are similar in symptoms. They have fevers, the flu, feel tired, become pale just like with leukemia…for example, we have a patient that was cured from *susto* at the same time she was receiving her cancer treatment, because they are completely different illnesses” (psychologist, psychoeducation).

## Discussion

In this study, caregivers of newly diagnosed children held varying and complex EMs of cancer that were elicited by an interdisciplinary team of clinicians throughout UNOP’s diagnostic process. As clinicians at UNOP walked caregivers through a new diagnosis of cancer, they prompted discussion about each component of the EM, allowing caregivers to share their own beliefs. In turn, clinicians were able to address misconceptions, provide culturally resonant explanations, and offer support to caregivers. To our knowledge, this is the first study applying the EM framework to pediatric cancer care, thus these findings provide insight into how this approach may be useful in pediatric cancer communication.

In LMICs, cancer treatment abandonment, defined as failure to start or complete treatment, has been linked to complex factors beyond socioeconomic status, including personal and cultural beliefs, prior experience with cancer, and miscommunication between clinicians and families [22, 24, 27, 35]. While there is a paucity of literature exploring EMs in pediatric oncology, research among global adult cancer populations has shown that a patient’s EM influences their participation in care and relationship with medical providers [1]. Therefore, the EM framework may be especially important to improve pediatric cancer outcomes in LMICs, where non-allopathic beliefs are prevalent [30, 34].

Clinical communication can be thought of as a transaction between patient and clinician EMs, where shared understanding emerges as the product of repeated clinical interactions during which EMs are disclosed and negotiated [2, 16, 20]. At UNOP, this unfolded throughout the diagnostic process as parental EMs were elicited and provider EMs were described. Caregivers arriving at UNOP had pre-existing EMs about cancer, however, similar to prior research among US-based adult populations with chronic illness, unprompted reference to *pathophysiology* was rare [3, 22]. Although *pathophysiology* may not have been a part of caregiver EMs upon arrival to UNOP, it was introduced by clinicians and accepted by caregivers. As has been demonstrated in other settings, sharing EMs can affect patient understanding of illness and engagement in therapy, as it allows healthcare providers to clarify misconceptions and share information to further their patients’ understanding of disease [3–6, 36].

Whereas clinicians in our study were more likely to employ *disease accounts*, caregivers used *illness accounts* to describe their initial understanding of cancer. Other work has similarly shown discrepancy between provider and lay models of disease in LMICs [37]. Importantly, clinicians at UNOP validated the co-existence of *illness* and *disease accounts* to explain a child’s illness course. Of note, UNOP clinicians were all native Guatemalans, thus their EMs may have aligned more closely with their patient population as compared to other cross-cultural contexts [11]. Previous research demonstrated that UNOP’s cancer education, which acknowledged pre-existing beliefs, led to improved parental treatment acceptance, further supporting how negotiating EMs can facilitate shared understanding [30]. While discrepancies between expectations for treatment can lead to abandonment, negotiating a common ground, where both *illness* and *disease* are addressed, may lead to improved treatment adherence and patient satisfaction [2, 27]. Further investigation of EMs in other settings, as well as comparison across settings, could provide valuable insight into how application of the EM framework may vary based on cultural context.

Although clinicians at UNOP were not utilizing the EM framework formally, this study demonstrates how interdisciplinary care supports the use of the EM. Other than *treatment*, all aspects of the EM were more likely to be discussed by and with the psychosocial team, stressing the importance of an interdisciplinary approach that capitalizes on different strengths and training. At UNOP, the implementation of the interdisciplinary team model led to a decrease in treatment abandonment from 21% to 7% in only seven years [28]. Integrated care models are associated with improved outcomes, and one mechanism may be through allowing the time and space to engage with patient and families’ pre-existing beliefs as was seen at UNOP [38]. Future work exploring the efficacy of using the EM model on clinical outcomes, such as uptake of medical care or satisfaction rates, is needed.

In recent years, medical education has increasingly emphasized culturally-competent care [10, 21, 39, 40]. Incorporating the EM into clinical practice is one approach. Several EM assessment tools have been created for use in research, however operationalization of EM frameworks into clinical settings has been limited [41, 42]. Kleinman proposed a series of questions that can be used during clinical encounters to elicit patient EMs [21]. Several iterations of Kleinman’s model have been adopted and additional approaches are undergoing study [40, 41, 43]. We propose that clinicians in all settings routinely ask patients and caregivers open-ended questions targeted to each component of the EM, particularly when discussing a new diagnosis, to promote shared understanding.

This study has several limitations. Whereas encounters were conducted in Spanish, data was analyzed in English, and there is potential for translation to have affected meaning. To address this, bilingual members of the research team reviewed all transcripts. Our study was located at one center in Guatemala. Despite being a small country, Guatemala is culturally and linguistically diverse, with twenty-four indigenous ethnic groups composing over forty-percent of the population [44]. While purposive sampling was used to recruit families of different backgrounds for the study, the findings may not fully capture the varied experience of all indigenous ethnic groups within Guatemala. In order to capture broader themes, analysis from this study was not stratified by diagnoses, however it is important to note that individual EMs may vary based on cancer diagnosis. Given the qualitative nature of this study, this analysis was intended to deeply explore the experiences of a small sample rather than produce generalizable results, therefore findings must be interpreted within the context of the study. Despite this, the conclusions drawn from this study may be transferable to other cross-cultural clinical settings. Further work is necessary to explore cross-cultural communication and investigate how the EM approach can be used in other culturally diverse, as well as non-cancer, pediatric settings.

## Conclusion

The present study employed an anthropologic lens to explore how UNOP’s model of care aligned with the EM framework. The insights gained through examining how clinicians at UNOP communicated a new cancer diagnosis while encouraging caregivers to discuss their EMs can be used to further our understanding of the role of communication in improving quality of care and outcomes for children with cancer [24, 25, 30]. The EM framework is applicable to all clinical interactions, but may be especially useful in cross-cultural encounters, where there is more likely to be misunderstanding and differences in beliefs. Moving forward, more research is needed to further operationalize the EM framework and study its impact in broader pediatric settings.

## Supporting information

S1 Checklist Inclusivity in global research. (DOCX)

S1 File The complete interview guide is available and provided on pages 2–3 of the supplementary material. (DOCX)
