# Supplementary material for: Explanatory models in real-world clinical interactions at a pediatric cancer center in Guatemala
Source: PLOS Glob Public Health. 2024 Oct 11;4(10):e0003813. doi: 10.1371/journal.pgph.0003813 (PMC11469487; doi:10.1371/journal.pgph.0003813)
Supplement: S1 File — (DOCX) [file pgph.0003813.s002.docx]

**Supplemental Material**

Explanatory models in real-world clinical interactions at a pediatric cancer center in Guatemala

Laura C. Harrison, MD, Silvia Rivas, MD, Lucia Fuentes, BA, Ana Cáceres-Serrano, PhD, Gia Ferrara, MSGH, Federico Antillon-Klussmann, MD, PhD, Carlos Rodriguez-Galindo, MD, Jennifer W. Mack, MD, MPH, Dylan E. Graetz, MD, MPH

| **Section** | **Page** |
| --- | --- |
| Interview Guide | 2-3 |

1. **Interview Guide**
2. Tell me about your experience at UNOP.
   1. Who told you to come?... how did you get here?... when did you arrive?... What happened next…?
      1. Where did you stay while your child was being diagnosed?
      2. Who came to the visits?
      3. Who visited you while you were here?
      4. Who did you meet with?
      5. What testing was done?
3. Before your child was diagnosed, what did cancer mean to you? What had you heard about cancer?
   1. Did you know anyone with cancer?
   2. How do people in your community think about cancer?
   3. Had you heard the word before? How did you first hear it/learn about it?
4. Tell me, did you go to another hospital or receive treatment anywhere before you came to UNOP?
   1. If so, where?
   2. What did they tell you about your child and his/her illness?
   3. Did you try any medicines or remedies before coming to UNOP?
      1. What happened with these?
5. At the time your child was diagnosed at UNOP, who explained cancer to you?
   1. How did they explain it?
   2. How was that similar to what you already understood/believed about cancer?
   3. How was it different to what you understood/believed about cancer?
   4. Did you talk to the team about these similarities/differences? Were all of your questions answered/addressed?
   5. How does this relate to your other experiences with illness?
      1. How is it similar/different?
6. What is your understanding of cancer now?
   1. How did you reach this understanding?
   2. Is this similar to or different from what your family thinks about cancer?
   3. Is it similar to or different from what others in your community think about cancer?
   4. Is it similar to or different from what the doctors and nurses think?
   5. Do you still have questions or concerns?
7. Tell me about how you usually make important decisions in your family/community.
   1. There are lots of decisions a family has to make, for example, some families have to make decisions about how to spend money or whether their children will work or go to school. Who is responsible for making decisions in your family?
      1. Are there others who have input in decisions?
      2. What is your level of involvement in decisions? Would you say you are mostly responsible for decisions alone? Do you share that responsibility? With whom? Do you have more limited input?
   2. How is this similar to or different from the way your family has made decisions about your child’s cancer?
      1. Who is responsible for coming to appointments with your child?
      2. How is information from those visits shared with others in your family? In your community?
      3. What do you need to help you make decisions about your child’s diagnosis and treatment?
      4. Does your child have a say in decisions regarding his or her care?
      5. Have there been disagreements about what to do for your child? Tell me more about those disagreements and how your family has handled that?
8. Now I would like to learn more about how you are feeling and what you are thinking about during this time, shortly after having a child diagnosed with cancer.
   1. Who supports you during this time?
   2. What changes have you had to make to your life/family?
   3. Have you felt supported by the team at UNOP? How, or how not? By whom?
   4. What are you worried about during this time? How does the staff at UNOP address these worries?
   5. What are you most hoping for during this time? How does the team at UNOP address these hopes?
   6. As you think about these hopes and worries for your child, which ones stand out as being the most important to you?
   7. How have your hopes and worries about other things in your life changed since having a child diagnosed with cancer?
9. If you had the opportunity now to speak with other parents of a child recently diagnosed with cancer, what would you tell them? What advice would you give them?
